# Theory-Based Interventions Combining Mental Simulation and Planning Techniques to Improve Physical Activity: Null Results from Two Randomized Controlled Trials

**DOI:** 10.3389/fpsyg.2016.01789

**Published:** 2016-11-16

**Authors:** Carine Meslot, Aurélie Gauchet, Benoît Allenet, Olivier François, Martin S. Hagger

**Affiliations:** ^1^TIMC-IMAG UMR CNRS 5525, Grenoble Alpes UniversityGrenoble, France; ^2^Laboratoire Interuniversitaire de Psychologie, Grenoble Alpes UniversityGrenoble, France; ^3^Pharmacy Department, Grenoble University HospitalGrenoble, France; ^4^Health Psychology and Behavioural Medicine Research Group, School of Psychology and Speech Pathology, Curtin University, PerthWA, Australia; ^5^Department of Sport Sciences, Faculty of Sport and Health Sciences, University of JyväskyläJyväskylä, Finland

**Keywords:** implementation intention, mental simulation, physical activity, behavior change intervention, health behavior

## Abstract

Interventions to assist individuals in initiating and maintaining regular participation in physical activity are not always effective. Psychological and behavioral theories advocate the importance of both motivation and volition in interventions to change health behavior. Interventions adopting self-regulation strategies that foster motivational and volitional components may, therefore, have utility in promoting regular physical activity participation. We tested the efficacy of an intervention adopting motivational (mental simulation) and volitional (implementation intentions) components to promote a regular physical activity in two studies. Study 1 adopted a cluster randomized design in which participants (*n* = 92) were allocated to one of three conditions: mental simulation plus implementation intention, implementation intention only, or control. Study 2 adopted a 2 (mental simulation vs. no mental simulation) × 2 (implementation intention vs. no implementation intention) randomized controlled design in which fitness center attendees (*n* = 184) were randomly allocated one of four conditions: mental simulation only, implementation intention only, combined, or control. Physical activity behavior was measured by self-report (Study 1) or fitness center attendance (Study 2) at 4- (Studies 1 and 2) and 19- (Study 2 only) week follow-up periods. Findings revealed no statistically significant main or interactive effects of the mental simulation and implementation intention conditions on physical activity outcomes in either study. Findings are in contrast to previous research which has found pervasive effects for both intervention strategies. Findings are discussed in light of study limitations including the relatively small sample sizes, particularly for Study 1, deviations in the operationalization of the intervention components from previous research and the lack of a prompt for a goal intention. Future research should focus on ensuring uniformity in the format of the intervention components, test the effects of each component alone and in combination using standardized measures across multiple samples, and systematically explore effects of candidate moderators.

## Introduction

Physical inactivity is related to all-cause mortality and implicated in 6% of total deaths globally (WHO, 2010a). Physical inactivity is also associated with increased risk from multiple chronic illnesses and conditions including cardiovascular disease, diabetes, some cancers, and obesity (Blair et al., 1995; Jeon et al., 2007; Bell et al., 2014; Brenner, 2014). Numerous studies have demonstrated the benefits of participating in regular physical activity. Population studies have shown that participation in regular physical activity is likely to reduced all-cause mortality, notably deaths caused by cardiovascular disease (Blair et al., 1995). This has led to the development of national guidelines for the type, frequency, intensity and duration of physical activity thought to confer health benefits. While there are idiosyncratic differences in guideline content, most advocate at least 30 min of moderate-to-vigorous physical activity on at least 5 days of the week (NHS, 2008; WHO, 2010b).

As a consequence many governments have aimed to develop national programs to intervene and promote participation in physical activity at the population level (Department of Health, Physical Activity, and Health Improvement and Protection, 2011). The development of such interventions, however, needs to be based on persuasive techniques and strategies to encourage individuals to take up physical activity and to provide them with the skills and personal resources to do so. Research on such techniques and strategies is based on formative psychological research that identifies the factors underpinning health behavior. At the forefront of this research are social cognitive theories derived from social psychology, which have provided insight into the factors that impact on decision making and have demonstrated efficacy in understanding the processes involved (Heckhausen and Gollwitzer, 1987; Conner and Norman, 2005; Hagger and Luszczynska, 2014). These factors can then be targeted in behavioral interventions using specific techniques to manipulate or change the influential factors (Biddle et al., 2007). Research, however, is needed to target and manipulate each specific component to fully understand the precise mechanisms underpinning behavior change. This can be done in factorial designs in which one or more intervention techniques can be targeted and their individual and interactive effects on a behavioral outcome evaluated (Peters et al., 2015). This is also important if the effectiveness of theories of behavior change in guiding behavior change interventions is to be evaluated.

Many social cognitive theories applied to predict and understand health behavior have intention as a focal variable and the most proximal predictor of behavior (Biddle et al., 2007; McEachan et al., 2011; Conner and Norman, 2015; Rich et al., 2015). However, research has consistently noted the generally weak link between intentions and behavior, known as the intention-behavior ‘gap’ (Orbell and Sheeran, 1998; Rhodes and de Bruijn, 2013; Sniehotta et al., 2014; Sheeran and Webb, 2016). According to Sheeran (2002), intention measures account only for 28% of the behavior. Furthermore, interventions focusing on changing interventions and its antecedents have been shown to be successful in changing intentions but are much less effective in changing behavior (Webb and Sheeran, 2006). Research has shown that some individuals can have strong intentions to engage in health behavior but experience difficulties in converting those intentions into action. These individuals have been identified as ‘inclined abstainers’ (Orbell and Sheeran, 1998) or ‘unsuccessful intenders’ (Rhodes and de Bruijn, 2013). Researchers have therefore sought to examine the process by which intentions are converted into action.

One approach has been to focus on volitional processes, that is, the processes involved in the enactment of intentions after they have been formed (Heckhausen and Gollwitzer, 1987; Schwarzer, 1992). Prominent among these is the model of action phases (Heckhausen and Gollwitzer, 1987; Gollwitzer, 1993), which makes the distinction between volitional and motivational phases prior to action. The motivational phase makes reference to the formation of actual intentions in which individuals form intentions based on their beliefs, similar to the process outline in the theory of planned behavior. The volitional phase occurs after the individual has formed and intention and made the decision to act. This phase outlines the process by which an individual enacts their intentions and is dependent on the individual developing a sufficient future strategy to enact the planned behavior. Whether individuals are successful in enacting a plan is, therefore, dependent on whether they have an intention to act and whether they have a sufficient strategy to carry out their intention. Accordingly, the model of action phases implies that the motivation is a necessary but not sufficient condition for behavioral enactment, which is where the intention-behavior gap arises. Intentions must, therefore, be augmented with sufficient strategies in the volitional phase that enables individuals to carry out their plans (Koestner et al., 2002, 2006, 2008; Sheeran et al., 2005; Chatzisarantis et al., 2010).

A prominent strategy that has been purported as a means to assist individuals in enacting their intentions and countering the gap between intention and behavior is implementation intentions (Sheeran and Webb, 2016). An implementation intention is a self-regulation strategy by which an individual augments their intention with a cue-initiated plan to increase the likelihood that they will carry out their intention. An implementation intention is a mental act linking an anticipated critical situation or ‘cue’ with the appropriate intended response (Gollwitzer, 1993). By identifying a cue present in the environment and pairing it with the intended action, the individual will increase the likelihood of the intention being enacted. The cue should be an appropriate condition, time and place in which the action should be performed. Gollwitzer (1993) suggests that the implementation intention leads to the establishment of a strong link between the cue and the goal-directed response. The mechanism by which implementation intentions exerts its effects is through the formation of effective plans at the post-intentional stage, i.e., rather than being mediated by intentions, implementation intentions should affect behavior through planning (Luszczynska, 2006). Meta-analyses of implementation intention interventions have shown a medium-to-large effect size in promoting health-related behaviors generally (Gollwitzer and Sheeran, 2006) and a small-to-medium effect size in promoting physical activity (Bélanger-Gravel et al., 2013). However, it is important to note that these meta-analyses included few unpublished studies and there are some indications of null or detrimental effects for implementation intention interventions in the literature (Jackson et al., 2005, 2006; Skar et al., 2011; Huang, 2012; Scholz et al., 2013; Jessop et al., 2014).

While implementation intentions have been found to be effective across studies, meta analyses have also identified considerable heterogeneity in the effects of implementation intention across the literature. Hagger et al. (2016) indicated that the way implementation intention manipulations are operationalized could potentially moderate their effect. The format and presentation of plans feature prominently as a potential moderator of planning interventions. Research has suggested that implementation intention effects are often facilitated by prompting individuals to form plans using and ‘if-then’ format, that is, prompting individuals to identify a critical situation and pair it with their intended behavior (Gollwitzer, 2014), as opposed to more ‘global’ or open-ended plans without an ‘if-then’ prompt (Chapman et al., 2009). Other critical moderators that have been identified include intrapersonal factors such as individual differences in planning capacity and time delay between the administration of the planning technique and the enactment of the target behavior. There have, however, been few systematic tests of these moderators using factorial designs in the literature (Hagger et al., 2016).

The effectiveness of implementation intentions is also dependent on individuals having sufficient motivation to engage in the behavior. Maximizing intentions, a construct that reflects an individual’s level of motivation toward a given behavior and the amount of effort they are prepared to invest in pursuing it, is an important prerequisite for the effectiveness of any planning intervention (Sheeran et al., 2005; Hagger and Chatzisarantis, 2009). This is based on the assumption in the model of action phases that volitional components like planning affect behavior in a post-decisional manner. Means to bolster motivation to participate in the behavior alongside a volitional strategy like implementation intentions, therefore, will ensure that an individual is an optimal motivational and volitional state to enact their intentions and engage in the behavior. An effective means to bolster motivation is through mental simulation. Mental simulation is a strategy in which individuals visualize or imagine engaging in the intended action (Pham and Taylor, 1999) and is supposed to reinforce individuals’ intention to act by increasing their propensity and the readiness to act (Pham and Taylor, 1999). As a consequence, the effects of mental simulation occur through changes in self-efficacy and it is considered to affect behavior as part of the motivational phase in Heckhausen and Gollwitzer’s (1987) model. Specifically, mental simulations boost self-efficacy as the imagined action serves as a ‘self-model’ or subjective ‘vicarious experience’ (Bandura, 1977). The effects are therefore expected to be mediated by self-efficacy perceptions. A sequential multiple mediation model can therefore be envisaged in which effects of mental simulations on action occur through changes in self-efficacy followed by changes in motivation or intention. There are two types of mental simulations. Outcome mental simulation requires individuals to imagine the achievement of the goal (e.g., simulating the situation once the goal is reached alongside the emotions and cognitions the individual is likely to have). Process mental simulation requires individuals to imagine the means required to attain the behavioral goal (e.g., simulating how the individual is enacting the behavior focusing on the details, like body sensations, emotions, details of the environment). Research on mental simulations has shown them to be effective in boosting behavioral engagement and goal-progress in many domains including health (Pham and Taylor, 1999; Vasquez and Buehler, 2007; Hagger et al., 2012a).

A further proposed mechanism for the effects of mental simulation on behavior change is through their capacity to ‘activate’ or make salient certain action-oriented mindsets or behavioral tendencies. Mental simulations may work by providing a bridge between thoughts and goal-directed behavior (Pham and Taylor, 1999). Mental representations of the goal-directed behaviors may involve fantasizing about past events that have already been experienced or future events that have never been realized. For example, studies have suggested that mental simulation activates an open-minded mindset while implementation intention activates an implemental mindset by focusing on the relevant components of if-then plans (Faude-Koivisto et al., 2009). But when mental simulation is realized before implementation intention, it would enable the individual to reflect on the different paths to set up a goal in the motivational phase of decision making and then set up strategies to achieve the goal in the volitional phase (Heckhausen and Gollwitzer, 1987).

Studies have investigated the effectiveness of combined mental simulation and implementation intentions interventions to promote reductions in alcohol consumption. The interventions were based on the hypothesis that combining a mental simulation intervention that promotes motivation with an implementation intention that promotes volition will optimize participation in health behavior (Knauper et al., 2011; Hagger et al., 2012a,b). This follows research that suggests that creating conditions that maximize intentions to engage in the health behavior and augmenting the intentions with means to implement the intentions should lead to optimal conditions for behavioral enactment (Prestwich and Kellar, 2014). A review of nine studies testing effects increasing intention strength and implementation intentions using full-factorial designs revealed that six showed significant interaction effect such that behavior participation was optimal when intentions were strengthened alongside implementation intentions. Hagger et al. (2012a) adopted a similar full-factorial design to examine the independent and interactive effects of mental simulations and implementation intentions on alcohol consumption. Results revealed that the combination of mental simulation and implementation intention interventions resulted in a significant decrease in the number of alcohol units consumed relative to participants receiving each of the intervention conditions alone or neither, but only among participants with high baseline alcohol consumption. Research adopting the same design has found that only implementation intention intervention was effective in evoking change (Hagger et al., 2012b). While these findings provide some indication that the combination of mental simulation and implementation intention strategies can lead to optimal engagement in health behavior, results do not provide unequivocal support and Hagger et al.’s (2012a) conditional findings for high alcohol consumers suggest that effects are most substantive when motivation is low and individuals are resistant to change and have high levels of an undesired behavior.

The model of action phases clearly dictates that motivation is a necessary but insufficient condition for behavioral enactment, and that behavioral enactment is also dependent on conditions in the volitional phase such as planning that lead to better behavioral enactment. Research has provided some support for the interactive effect of motivational and volitional interventions in promoting health behavior, including mental simulations with implementation intentions (Milne et al., 2002; Hagger et al., 2012a), but some research has not supported the interactive effect. These may be findings that are specific to a particular behavior or particular context, or, as in the Hagger et al. (2012b) study, among participants with high levels of the undesirable behavior to be changed. In the present study we aimed to conduct a series of interventions using mental simulations and implementation intentions to promote physical activity behavior. In Study 1, we examined the effect of an intervention combining implementation intentions and mental simulations on physical activity participation relative to an implementation intention intervention alone and a no intervention control condition in a student sample. We expected that the combined intervention condition would yield greater participation in physical activity relative to the implementation intention only condition and the control condition after controlling for baseline physical activity. We also expected participants in the implementation intention condition to report greater physical activity participation than those in control condition. In Study 2 we examined the unique and interactive effects of these manipulations on physical activity using a full-factorial design. In addition, we aimed to provide a robust test of the interactive effects of these interventions by examining their effects in a participants including students and members of the general public joining a fitness center with little or no previous experience of physical activity and using center attendance as an objective proxy measure of physical activity participation. We expected to find main effects of the mental simulation and implementation intention interventions on physical activity participation post-intervention controlling for baseline values on physical activity. We also expected to find an interaction effect of the mental simulation and implementation intention interventions such that participants receiving both intervention would exhibit the highest levels of physical activity participation relative to each condition alone and a control condition receiving neither.

## Study 1

Adopting a three-group cluster-randomized design, we aimed to examine the effects of a mental simulation and implementation intention intervention on physical activity behavior in a sample of students relative to an implementation intention only intervention and a measurement-only control group (Supplementary File). The intervention conditions and measures of behavioral and psychological variables were administered at baseline (T1) with 1-week (T2)^1^ and 4-week (T3) behavioral follow-up occasions. We expected that the participants who formed a mental simulation alongside an implementation intention (mental simulation plus implementation intention group) would report higher levels of physical activity at 1 and 4 weeks post-intervention than participants who formed an implementation intention (implementation intention group) alone and participants who received neither of the intervention components (control group).

### Materials and Methods

#### Participants

Participants were undergraduate students from Grenoble Alpes University (*N* = 92, females = 79, males = 13; *M*_age_ = 24.4 years, *SD* = 6.44) recruited on a voluntary basis by advertising the study to students eligible to participate before their classes. Students were considered ineligible to participate if they were aged less than 18 years or had reduced mobility or a disability which prevented them from participating in physical activity.

#### Design and Procedure

At baseline, all participants completed consent forms followed by a baseline questionnaire containing self-report measures of demographic details (age, BMI, smoking status, number of alcoholic drinks consumed per occasion and frequency of alcohol consumption) (Dollinger and Malmquist, 2009; Hagger and Montasem, 2009), theory of planned behavior variables (attitude, subjective norms, perceived behavioral control and intention) (Ajzen, 1991) and past physical activity behavior (Conner and Armitage, 1998). Participants allocated to the implementation intention group were then prompted to form an implementation intention with respect to physical activity presented as a pen-and-paper exercise. Participants allocated to the mental simulation plus implementation intention group formed a mental simulation, again as a pen-and-paper exercise, prior to forming an implementation intention. Participants from the two experimental groups then completed manipulation check measures of mental simulation and/or implementation intentions according to their allocated group. Participants allocated to the control group completed the measures but did not receive any intervention. One and 4 weeks after baseline, participants completed follow-up self-report measures of theory of planned behavior variables, and physical activity behavior. Three teachers from the Grenoble Alpes University were contacted to give them information about the study and ask for their authorisation to recruit students from their classes. We chose to randomize the sample by clusters determined by university class using a random numbers table in order to reduce the likelihood of data contamination through participants allocated to different conditions conferring. Regarding the sequence generation, no allocation concealment was made. Patients were blinded to group allocation, but the experimenters administering the study materials were not. Data were collected from March to May 2015 and we stopped collecting the data when sufficient numbers of participants were included to achieve adequate statistical power.

#### Informed Consent

At baseline, participants read an information sheet describing the study and outlining their rights and benefits, and the potential risks of participation. They then signed a written informed consent form which was detached from the questionnaire thereafter to maintain participant anonymity. At the three data collection occasions, participants formed a unique identifier comprising the first two letters of their mother’s name, father’s name, and their birth month and day. Questionnaires were matched across data collection points using participants’ unique identifier. Prior to data collection ethical approval was obtained from the institutional review board of the University of Grenoble.

#### Implementation Intention Intervention

Participants allocated to the implementation intention condition and to the mental simulation plus implementation intention condition were asked to form implementation intentions with respect to regular physical activity for the forthcoming month. An ‘if-then’ format was used to link the situation (preceded by the prompt “if”) with the goal-oriented behavior (preceded by the prompt “then”) (Gollwitzer, 1993). Participants were presented with the following script, which was followed by a series of prompts including the day and hour the participant planned to exercise, the type of exercise, and the place where they planned to exercise accompanied by a series of blank lines so that they could write down their responses:

“A lot of people have the intention to be more physically active, but they forget or do not find the time to do it. Some studies have shown that if you form a plan specifying when, where and how you will do your physical activity, you will be more likely to remember to do it and find the time to do it. Thus, it would be useful to spend a few minutes to think about the times when you will participate to a physical activity in the next month. Try to think about the best time for you to exercise, and do not share your answers with your colleague.”

#### Mental Simulation Intervention

Participants in the mental simulation plus implementation intention condition were prompted to carry out a mental simulation exercise prior to the formation of an implementation intention. Our mental simulation manipulation was identical to that used by Pham and Taylor (1999) adapted for physical activity and focused on process mental simulation. Participants were presented with the following written message, which was followed by a series of blank lines on the page for participants to write down their response:

“In this exercise, you will be asked to visualize yourself doing physical activity to increase your regular participation in physical activity. As of today, and for the forthcoming month, imagine how you would do physical exercise regularly. It is very important that you actually ‘see’ yourself doing physical activity and have that picture in your mind. Please write on the lines below how you imagine you will achieve your goal of doing regular physical activity, in the following month.”

### Measures

#### Theory of Planned Behavior Variables

We measured the variables related to the theory of planned behavior (Ajzen, 1991) adapted to refer to regular participation in physical activity. Intention to engage in physical activity was measured using four items (e.g., “I intend to do physical activity regularly in the forthcoming month”). Attitudes toward physical activity were measured using five items (e.g., “For me doing physical activity regularly in the forthcoming month is harmful/beneficial”). Subjective norms (e.g., “Most people who are important to me think that I should/I should not do physical activity regularly”) and the probability the participant would enact the behavior (e.g., “It is expected of me that I do physical activity regularly in the forthcoming month”) were measured using four items each. Perceived behavioral control as function of the perceived difficulty (e.g., “For me doing physical activity regularly in the forthcoming month would be impossible/possible”) and ability (e.g., “If I wanted to, I could practice physical activity regularly in the forthcoming month”) to engage in the behavior were measured using two items each, the total score for each was computed with the mean of the two items. All the responses were made on 7-point Likert scales, with the exception of the attitudes scale which was measured on 7-point bi-polar semantic differential scales.

#### Physical Activity Behavior

The International Physical Activity Questionnaire – Short Form (IPAQ-7; Craig et al., 2003) was used to measure self-reported physical activity. Participants indicated the number of minutes, hours, or days during which they did physical activity over the previous seven last days at three levels of intensity: vigorous activity, moderate physical activity, and walking. Each of level of intensity was accompanied by a detailed description so that participants were familiar with the types of activity that fell into each category. The measure yields a total physical activity score expressed in Metabolic Equivalent Task per minute (METs/min), an indicator of energy expenditure as a function of physical activity intensity.

#### Manipulation Checks

Participants in the intervention groups also completed manipulation check measures (Hagger et al., 2012a) to measure the extent to which they adhered to the instructions for the mental simulation (e.g., “To what extent have you figured out exactly how you might do physical activity regularly over the next month”) and implementation intention (e.g., “To what extent do you have a plan for when, where, and how you might do regular physical activity in the following month”) exercises on 7-point Likert scales (from 1 “I have no idea” to 7 “I have figured out exactly”).

### Results

#### Participants

**FIGURE 1:**
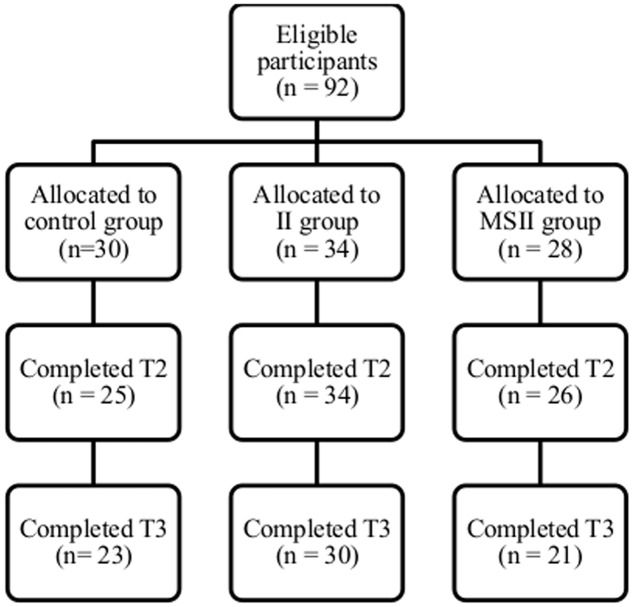
**Flow chart of participants from Time 1 to Time 3 of the 4-week follow-up (Study 1)**.

Ninety-two students agreed to participate and completed study measures questionnaires at T1 (females = 79, males = 13; *M*_age_ = 24.4 years, *SD* = 6.44). Seven participants (five in the control group and two in the mental simulation plus implementation intention group; 7.61% of the initial sample) dropped out of the study at T2 and 11 participants (two in the control group, four in the implementation intention group and five in the mental simulation plus implementation intention group; 9.47% of the initial sample) dropped out of the study at T3 (**Figure 1**). Of the recruited participants, five dropped out of the study at T2 only (three in the control group and two in the mental simulation plus implementation intention group), 15 dropped out at T3 only (five in the control group, three in the implementation intention group and seven in the mental simulation plus implementation intention group) and two dropped out at both T2 and T3 (all in the control group). Thus 64 participants (58.88% of retention rate) completed the study at the 2- and 4-week follow-up occasions (females = 59, males = 10; *M*_age_ = 25.19 years, *SD* = 5.44).

#### Missing Data

Preliminary analyses of the raw data from the questionnaire measures at follow-up revealed that 7.12% of the data values were missing due to failures to respond. Little’s MCAR test revealed that values were missing completely at random (χ^2^ = 163.352, df = 175, *p* = 0.726). Missing data were imputed using multiple imputation in SPSS v. 20 using the fully conditional specification imputation method with 10 iterations and a linear regression model for scaled variables (SPSS Inc.).

#### Attrition Checks

Chi-square tests and MANOVAs were used to test for differences on study variables due to attrition across the time points. Differences on categorical variable (i.e., gender) were tested using a χ^2^ analysis with the level of the variable of interest (male vs. female) cross-tabulated with attrition (dropped out of study after T1 vs. remained in study at T3). Differences on continuous demographic (age, BMI, alcohol consumption) and psychological (theory of planned behavior, self-efficacy) and behavioral (physical activity) variables were tested using MANOVAs with the study variables as multiple dependent variables and attrition as the independent variable. Participants who completed the study did not differ significantly from the participants who dropped out on gender [χ^2^(1, *N* = 92) = 0.03, *p* = 0.863, $\eta_{p}^{2}$ = -0.018] and on demographic, psychological and behavioral variables [Wilks’ Λ = 0.87, *F*(9,75) = 1.25, *p* = 0.277, $\eta_{p}^{2}$ = 0.131].

#### Randomisation Checks

Sample demographic and psychological data are presented in **Table 1**. Randomisation checks were conducted using χ^2^ tests and a MANOVA. There was no significant multivariate effect for intervention condition on the demographic, psychological and behavioral variables at baseline, but the effect only narrowly fell short of conventional levels of statistical significance [Wilks’ Λ = 0.69, *F*(18,148) = 1.66, *p* = 0.052, $\eta_{p}^{2}$ = 0.168]. When we looked at the between-participants effects, the three groups differed significantly on the physical activity measure at baseline [*F*(2,82) = 3.80, *p* = 0.026, $\eta_{p}^{2}$ = 0.085]. In addition, there were fewer smokers in the mental simulation plus implementation intention group (3 smokers) than in the implementation intention group (15 smokers) and in the control group [14 smokers; χ^2^(2, *N* = 91) = 10.94, *p* = 0.027, $\eta_{p}^{2}$ = 0.245]. There were also more men in the mental simulation plus implementation intention group (eight males) compared to the implementation intention (three males) and control (two males) groups [χ^2^ (2, *N* = 92) = 6.98, *p* = 0.031, $\eta_{p}^{2}$ = 0.275].

**Table 1:** Self-reported sample characteristics of Study 1 (*n* = 92).

	Control group (*n* = 30)	Implementation intention group (*n* = 34)	Mental simulation plus implementation intention group (*n* = 28)
**Age (years)**			
Mean (*SD*)	24.75 (6.04)	23.21 (5.03)	25.87 (8.63)
**Gender**			
Women	28 (35.4%)	31 (39.2%)	20 (25.3%)
Men	2 (15.4%)	3 (23.1%)	8 (61.5%)
**BMI**			
Mean (SD)	21.49 (2.30)	21.94 (2.65)	21.33 (3.54)
**Smoking status**			
Smoker	14 (43.8%)	15 (46.9%)	3 (9.4%)
Non-smoker	15 (28.8%)	17 (32.7%)	20 (38.5%)
Former smoker	1 (14.3%)	2 (28.6%)	4 (57.1%)
**Number of alcoholic drinks per week**			
Mean (SD)	2.68 (1.22)	3.09 (1.40)	2.26 (1.54)
**Frequency of alcohol consumption**			
Mean (SD)	2.14 (1.46)	1.74 (1.08)	1.78 (1.68)
**Attitude**			
Mean (SD) at T1	5.90 (0.79)	5.58 (1.53)	5.81 (1.17)
Mean (SD) at T2	5.87 (0.85)	5.79 (1.14)	6.03 (1.01)
Mean (SD) at T3	5.99 (0.95)	5.80 (1.24)	5.90 (1.47)
**Subjective norms**			
Mean (SD) at T1	4.85 (0.81)	4.38 (0.96)	4.78 (1.10)
Mean (SD) at T2	4.59 (0.89)	4.16 (1.18)	4.43 (1.32)
Mean (SD) at T3	4.61 (0.94)	4.14 (1.27)	4.24 (0.95)
**Intention**			
Mean (SD) at T1	4.92 (2.01)	5.35 (1.79)	4.70 (2.17)
Mean (SD) at T2	5.00 (1.77)	5.36 (1.66)	4.92 (1.93)
Mean (SD) at T3	5.04 (1.78)	5.00 (1.88)	4.91 (1.97)
**Perceived behavioral control**			
Mean (SD) at T1	4.88 (1.46)	4.63 (1.57)	4.52 (1.51)
Mean (SD) at T2	4.63 (1.40)	4.47 (1.68)	4.43 (1.63)
Mean (SD) at T3	4.58 (1.56)	4.20 (1.83)	4.80 (1.61)
**MET-min per week**			
Mean (SD) at T1	7.38 (1.18)	7.57 (0.85)	6.90 (1.52)
Mean (SD) at T2	7.49 (0.87)	7.37 (1.49)	7.21 (1.30)
Mean (SD) at T3	7.68 (0.86)	7.64 (0.98)	7.35 (1.17)


#### Intervention Effects

The primary physical activity outcome variable expressed in MET-min did not follow a normal distribution, so we computed a logarithmic transformation. We opted for a natural logarithmic transformation, because the data were calculated on a proportional basis and were easily interpretable (Gelman and Hill, 2007). Physical activity data for the intervention conditions are presented in **Table 2**.

**Table 2:** Results of linear mixed effect model of physical activity measured at T2 and T3 on interventions (Study 1).

	Mean between-group difference^a^	Standard deviations^b^	Cohen’s *d*^c^	*t*-value	*p*-value
**Fixed effects**					
Implementation intention only group vs. control group	-0.28	0.26	-0.38	-1.10	0.27
Mental simulation plus implementation intention group vs. control group	0.07	0.27	0.10	0.25	0.79
Mental simulation plus implementation intention group vs. Implementation intention only group	0.36	0.23	0.49	1.53	0.13
Residual		0.73			
AIC	537.69	-		-	-
Log-likelihood	-260.85	-		-	-


*Linear mixed effect model based on 92 participants and 276 observations. Dependent variable in all cases in metabolic equivalent (MET) per minute (MET.min^-1^). AIC, Akaike Information Criterion.*

*^a^Difference in mean MET.min^-1^ across intervention groups at T2 and T3 combined.*

*^b^Standard deviations of effect sizes estimates.*

*^c^Cohen’s *d* effect size coefficients were computed using residual standard deviations.*

Given the risk of contamination related to the cluster design and the three-wave design, we tested our hypotheses using a regression model with random effects using recursive maximum-likelihood estimates and using R version 3.1.1 with the “nlme” package (Lindstrom and Bates, 1988). We analyzed the effect of the intervention by conducting a linear mixed effect model regression of the intervention conditions on physical activity measured at T2 and T3 controlling for physical activity measured a T1. A dataset was created for the mixed model with the three groups (control group, implementation intention group and mental simulation and implementation intention group) as between-participants effects and time as a within-participants effect on the physical activity scores at each time-point. Model selection based on Akaike’s information criterion selected a model with a random slope. The resulting effects indicate between-group comparisons Cohen’s *d* effect size coefficients were computed using residual standard deviations (Rouder et al., 2012). Results are presented in **Table 2**. Results revealed no significant between-group effects on aggregate values for on physical activity measured in MET-min per week at T2 and T3, controlling for gender (as the groups were not equivalent on the males percentage) and physical activity at T1^2^. However, effect size statistics suggested medium effect sizes for the between-group comparisons for the combined group, which implies that we had insufficient statistical power to detect these effects.

### Discussion

The purpose of the current study was to examine the effects of an intervention combining mental simulation and implementation intention strategies to promote physical activity at 1- and 4-week post-intervention follow-up. We conducted the intervention in a sample of undergraduate students adopting a three-group cluster-randomized design with classes allocated to an implementation intention group, a mental simulation plus implementation intention group, or a control group. Findings revealed no differences in physical activity participation at follow up between the implementation intention, implementation intention plus mental simulation, and control groups. While these data indicated that these intervention techniques were not effective in promoting physical activity participation in this study and were inconsistent with the bulk of the research on implementation intention and mental simulation in research on health behavior, the effect size data indicated that possible differences were present but the study was underpowered. It is, therefore, important to interpret current findings in the context of its limitations and acknowledge the importance of replication in larger, representative samples.

The current study had some limitations. The lack of provision of a goal intention in advance of completing the implementation intention exercise meant that the implementation intention exercise would have had very little relevance to those who did not have a goal of increasing their physical activity participation. The adoption of a cluster randomisation design may not have led to homogenous groups on key demographic and behavioral variables, although we controlled for potential variations on gender distribution, smoking status, and the number of alcoholic drinks per week to counteract differences across clusters. The reliance on a self-report measure of physical activity despite acceptable psychometric properties of the IPAQ-7 measure and good correlations with objectives measures in previous research (Craig et al., 2003) was a potential source of error variance. However, the most prominent limitation was the small sample size and the medium-sized effect sizes seemed to indicate this despite the non-significant findings. As a consequence, the current effect deserves further replication in a sufficiently powered trial. A final limitation was the lack of a full-factorial randomized controlled design enabling us to study the main effects of each intervention component, mental simulation and implementation intentions, independent of the interaction effect. This is important if researchers are to decode the precise components responsible for affecting a change in physical activity behavior, and whether the interactive effects are truly separate from the main effects of each component.

## Study 2

Study 2 added to and extended the findings of Study 1 by adopting a full-factorial, fully randomized between-participants design with a larger sample. Study 2 also had the advantage of recruiting participants who had signed up to attend a fitness center and had not attended the center previously, who were low active, and had no recent experience of physical activity behavior (Supplementary File). Two weeks after their enrolment to the fitness center (Time 1, T1), they were randomly allocated to mental simulation, implementation intention, mental simulation plus implementation intention group, or control groups. In addition, our target dependent variable was fitness center attendance verified through participants’ fitness center records and used as a proxy measure of physical activity participation, with 4-week (Time 2, T2) and 19-week (Time 3, T3, only for a subsample) behavioral follow-up occasions. We expected that participants who received the mental simulation and implementation intention manipulations together would exhibit higher fitness center attendance at follow-up than participants receiving the mental simulation and implementation intention interventions alone. We also expected participants receiving either the mental simulation manipulation, implementation intention manipulation, or both, to have greater attendance at the center than participants in the control group that received neither of the manipulations.

### Materials and Methods

#### Participants

Participants (*N* = 184, females = 97, males = 87; *M*_age_ = 26.95 years, *SD* = 9.59) were recruited from two different locations and randomized to each intervention conditions within each location, on a voluntary basis. In the first sample, participants were recruited from the University of Essex fitness center, which had both student and non-student attendees. In the second sample, participants were recruited from a commercial fitness center in the city of Stockport, UK, which comprised individuals from the general population. As the two studies adopted the same design and used the same materials and protocol, we opted to combine the samples. Although this decision violated randomisation for the overall sample, randomisation was retained within the samples (see Results section), and our decision to pool the samples was based on strict criteria to ensure there were no differences in the samples on key demographic factors. Inclusion criteria were identical to those of Study 1 except that participants aged under 18 years were also eligible for inclusion in the study as they had passed the fitness center induction criteria.

#### Design and Procedure

A randomized controlled design was adopted with four groups: implementation intention only, mental simulation only, mental simulation plus implementation intention, and control groups. The design was identical in both samples. Statistical power analysis revealed that a sample size of 144 participants was required (power = 0.95, alpha = 0.05, *d* = 0.65, *f* = 0.325; Gollwitzer and Sheeran, 2006) for a two-way mixed-model ANOVA with time [with two levels: baseline (T1), 4-week follow-up (T2) or 19-week follow-up (T3)] as the within-participants factor and experimental condition (condition: implementation intention only, mental simulation only, mental simulation plus implementation intention group, control group) as the between-participants factor. The participants were asked to participate at T1 in the 2nd week after their enrolment to the centers. They were randomly allocated to the mental simulation plus implementation intention group, the implementation intention group, the mental simulation group, or the control group within each sample.

At baseline, all participants completed measures of socio-demographic, past behavior and theory of planned behavior variables (i.e., attitude, intention, subjective norms, and perceived behavioral control). Consistent with the findings of Study 1, BMI, smoking status, alcohol consumption and self-control were excluded as these measures had no relation to the behavioral outcome and we wanted to reduce response burden on participants.

At T1, participants were allocated to the mental simulation plus implementation intention group, the implementation intention group, the mental simulation group or the control group using a random numbers table generated by the experimenter. No allocation concealment was made regarding the sequence generation. Patients were blinded to group allocation, but the experimenter administering the study materials was not. The participants were allocated to the implementation intention group were prompted to form implementation intentions and participants allocated to the mental simulation group were required to undertake a mental simulation exercise. Participants from the mental simulation and implementation intention group were required to undertake both exercises. Participants allocated to the control group completed the demographic, psychological, and physical activity measures only. At T2, all participants completed follow-up measures of theory of planned behavior variables. Participants from University of Essex campus fitness center also completed these measures at T3 because we were unable to obtain further access to the participants from Stockport fitness center at T3. Fitness center attendance was measured at the three time points, 2 weeks after initial enrolment to the fitness center (T1), at a 6-week follow-up occasion (T2) for both samples, and at a 19-week follow-up (T3) for participants from University of Essex campus fitness center. Participants were contacted via email with their email address and participant code stored in a database separate to the main data with data subsequently matched by a researcher who did not have access to the participants’ data to retain participant anonymity. Data were collected between September and April, 2008. We stopped collected the data when sufficient numbers of participants were recruited according to our power analysis.

#### Informed Consent and Anonymity

Participants read an information sheet describing the study and outlining the rights, benefits, and potential risks of the study to participants before being enrolled in the study. They then signed a written informed consent form which was detached from the questionnaire thereafter in order to maintain participant anonymity. A unique identifier comprising the first two letters of participants’ home post code, the first three letters of their mother’s maiden name and their landline phone number was formed at each time point of the study. This study obtained the ethical approval from the University of Essex Department of Psychology Research Ethics Committee prior to data collection.

#### Intervention Manipulations

Before the mental simulation and implementation intention statements, the intervention groups read the following introductory passage:

“Please complete the following mental and/or visualization exercises, they will only take about 5 min of your time. Please take care to complete each exercise in full and follow the instructions carefully, they will ultimately benefit you in future.”

In addition, participants were provided with WHO guidelines for physical activity at the beginning of the questionnaire using the following passage: “3–4 periods of exercise per week lasting no less than 30 min.” The intervention was a pen-and-paper exercise and participants were not assisted in performing the mental simulation and implementation intention exercises.

#### Implementation Intention Manipulation

Participants allocated to the implementation intention condition or mental simulation plus implementation intention condition were prompted to form an implementation intention with respect to their fitness center (gym) attendance. They were presented the following script which was followed by a series of blank lines to write down their plan:

“You are more likely to carry out your intention to use the gym regularly if you make a decision about where and when you will do so. Decide now when and how you will use the gym over the following weeks. You may find it useful go to the gym just before or just after something else that you do regularly, such as lectures. Please write in below when and how you will use the gym in the next 6 weeks (e.g., ‘I will do the ‘Fat Burning’ class on every evening after my lecture’).”

#### Mental Simulation Intervention

Participants in the mental simulation condition and mental simulation plus implementation intention condition performed the mental simulation exercise. We used the same manipulation as Pham and Taylor (1999) adapted to physical activity with some minor modification to encourage individuals to engage in the visualization exercises and focusing on the outcomes. They were presented with the following text introducing the exercise:

“Think about the exercise related goal that you have just decided on. Visualize yourself having achieved that goal. You have put a lot of effort into the achievement of your task and you have finally accomplished it. Imagine the effort you have made. See yourself standing at the point of success from where you look back on the work you did to get there. Imagine how your life is different since you started exercising regularly. Visualize the changes that resulted from the accomplishment of this goal. How does it feel to have started a new lifestyle that is good for you? Picture your life as it is now. Concentrate the on the feelings that you have when you do something that is really good for you. Visualize the satisfaction you feel at having achieved your goal. Picture the pride you feel, the confidence you feel in yourself, knowing you were successful. Try to feel the satisfaction you have with this accomplishment. Feel how proud and confident you are. Think about you daily routine. What does your day look like, now that exercising is a firm part of it? Imagine a typical day and see yourself engaging in your new exercise routine. See yourself standing at the point of success. Picture yourself thinking back to when you started working on your goal. How do you feel having successfully accomplished what you wanted? Concentrate on the energy that your healthier lifestyle contributes to your life. How does it feel to have more energy, more confidence, and to know that you successfully started a new healthier lifestyle?”

### Measures

#### Theory of Planned Behavior Variables

Measures of the theory of planned behavior constructs were similar to those used in Study 1 with the exception that they made reference to fitness center (gym) attendance. Intention to engage in physical activity was measured using three items (e.g., “I intend to to use the gym 3–4 days a week over the next 6 weeks”). Attitudes toward physical activity were measured using six items (e.g., “For me, using the gym 3–4 days a week over the next 6 weeks is harmful/beneficial”). Subjective norms (e.g., “Most people who are important to me would want me to use the gym 3–4 days a week over the next 6 weeks”) and the likelihood the participant would enact the behavior (e.g., “Most people close to me expect me to use the gym 3–4 days a week over the next 6 weeks”) were measured using four items. Our measure of perceived behavioral control comprised six items relating to perceived control (e.g., “How much personal control do you have in using the gym 3–4 days a week over the next 6 weeks?”) and ability (e.g., “I believe I have the ability to use the gym 3–4 days a week over the next 6 weeks”) with respect to engaging in the behavior. All the responses were made on 6-point Likert scales, with the exception of the attitude scale which was measured on 6-point bi-polar semantic differential scales.

#### Past Behavior

Self-reported past physical activity behavior was measured using a single item (“How often in the past have you used a gym?”) with responses provided on a 6-point Likert scale ranging from 1 (never) to 6 (always). This single item measure is derived from Godin and Shephard (1997) leisure-time exercise questionnaire and has been used in previous studies (Pihu et al., 2008).

#### Fitness Center Attendance

We measured participants’ frequency of attendance at the fitness center at a 6-week follow-up time point post-intervention (T2) as well as a 19-week follow-up time point (T3) for the University of Essex participants. Attendees were issued with a unique coded pass card that they were required to swipe on an electronic card reader on each occasion they attended the fitness center. Attendance data for each participant was automatically logged and stored electronically. Participating fitness centers provided the researchers with an anonymised list of participants’ attendance records using the separate list of codes and participant names provided by researchers. This ensured that study data was never matched with participant names to retain anonymity. Participants consented to having their fitness center attendance data provided to the research team prior to signing their consent form.

### Results

#### Participants

**FIGURE 2:**
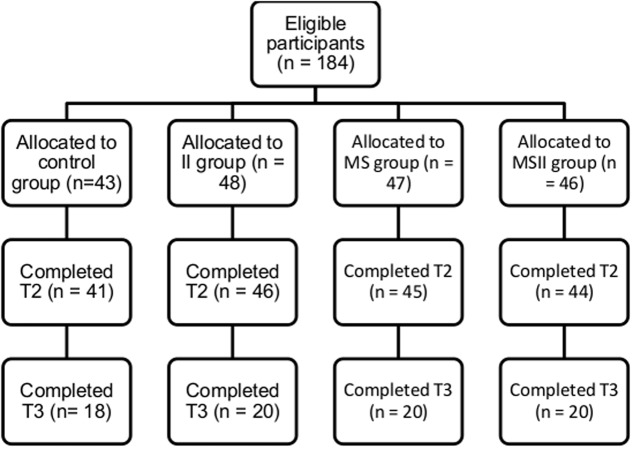
**Flow chart of participants from Time 1 to Time 3 of the 19-week follow-up (Study 2)**.

One hundred and eighty-four participants completed the questionnaires at T1 (females = 87, males = 97; *M*_age_ = 26.95 years, *SD* = 9.59). Eight participants (two participants in each group; 7.61% of the initial sample) dropped out of the study at T2 leaving 178 participants (94.57% of response rate) remaining in the study at 6-week follow-up (females = 93, males = 83; *M*_age_ = 27.15 years, *SD* = 9.72). At T3, seventy-eight participants (42.39% of the initial sample) from the sample from the University of Essex campus fitness center were contacted to complete the 19-week follow-up measures (females = 48, males = 20; *M*_age_ = 21.81 years, *SD* = 3.93; **Figure 2**).

.

#### Preliminary Analyses

##### Missing data

Preliminary analyses revealed that 5.44% of the data values were missing at follow-up due to response failures and Little’s MCAR test revealed that values were not missing completely at random (χ^2^ = 113.68, df = 21, *p* < 0.001). Missing data were imputed using multiple imputation using the same parameters as Study 1.

##### Attrition checks

A χ^2^ analysis revealed that participants who completed the study did not differ significantly from participants who dropped out on gender [χ^2^(1, *N* = 184) = 0.03, *p* = 0.875, $\eta_{p}^{2}$ = 0.012]. A MANOVA was used to test the differences on continuous demographic (i.e., age), psychological (theory of planned behavior), and behavioral (physical activity) variables by attrition rate. The MANOVA revealed an overall statistically significant multivariate effect for attrition [Wilks’ Λ = 0.929, *F*(6,177) = 2.24, *p* = 0.042, $\eta_{p}^{2}$ = 0.071]. Univariate tests showed that participants who completed the study had a higher fitness center attendance at baseline [*F*(1,182) = 8.14, *p* = 0.005, $\eta_{p}^{2}$ = 0.043] and a higher perceived behavioral control [*F*(1,182) = 10.14, *p* = 0.002, $\eta_{p}^{2}$ = 0.053] compared to participants who dropped out. As a consequence, we controlled for these variables in the main analyses.

##### Randomisation checks

Sample demographic and psychological data are presented in **Table 3**. Randomisation checks, conducted using χ^2^ tests and a MANOVA, showed no differences across intervention conditions on gender distribution [χ^2^(3, *N* = 184) = 3.92, *p* = 0.270, $\eta_{p}^{2}$ = 0.146] and demographic, psychological and behavioral variables [Wilks’ Λ = 0.92, *F*(18,495) = 0.79, *p* = 0.719, $\eta_{p}^{2}$ = 0.026; **Table 3**], for the overall sample. With reference to the first sample, there were also no differences in conditions on gender [χ^2^(3, *N* = 76) = 1.72, *p* = 0.633, $\eta_{p}^{2}$ = 0.150] and the demographic, psychological and behavioral variables [Wilks’ Λ = 0.71, *F*(21,190) = 1.15, *p* = 0.300, $\eta_{p}^{2}$ = 0.108]. In the second sample, no differences in condition were found on the demographic, psychological and behavioral variables [Wilks’ Λ = 0.75, *F*(21,281) = 1.39, *p* = 0.119, $\eta_{p}^{2}$ = 0.090]. However, there were significantly fewer males in the mental simulation group (10 males) than in the control (15 males), implementation intention (20 males), and mental simulation plus implementation intention groups [18 males; χ^2^(3, *N* = 108) = 8.37, *p* = 0.039, $\eta_{p}^{2}$ = 0.278]. These data provided good indication that there were no variations across samples from the two locations due to randomisation, and given the identical study design, we pooled data from both locations.

**Table 3:** Sample characteristics in Study 2 (*n* = 184).

	Control group (*n* = 43)	Implementation intention group (*n* = 48)	Mental simulation group (*n* = 47)	Mental simulation and implementation intention group (*n* = 46)
**Age (years)**				
Mean (SD)	27.74 (7.88)	27.13 (10.66)	26.26 (9.93)	26.72 (9.77)
**Gender**				
Women	22 (22.7%)	21 (21.6%)	30 (30.9%)	24 (24.7%)
Men	21 (24.1%)	27 (31%)	17 (19.5%)	22 (25.3%)
**Attitude**				
Mean (SD) at T1	4.74 (0.74)	4.69 (0.66)	4.68 (0.66)	4.71 (0.81)
Mean (SD) at T2	4.78 (0.69)	4.81 (0.52)	4.74 (0.66)	4.77 (0.75)
**Subjective norms**				
Mean (SD) at T1	4.50 (0.82)	4.25 (1.01)	4.32 (0.88)	4.42 (0.79)
Mean (SD) at T2	4.35 (0.94)	4.24 (0.87)	4.53 (0.84)	4.45 (0.71)
**Intention**				
Mean (SD) at T1	4.44 (1.12)	4.16 (1.01)	4.34 (0.74)	4.41 (0.95)
Mean (SD) at T2	4.58 (1.14)	4.21 (0.98)	4.58 (1.08)	4.34 (0.92)
**Perceived behavioral control**				
Mean (SD) at T1	4.46 (0.79)	4.46 (0.76)	4.41 (0.63)	4.45 (0.72)
Mean (SD) at T2	4.51 (0.68)	4.49 (0.77)	4.49 (0.71)	4.52 (0.69)


##### Intervention Effects^3^

A 2 (mental simulation: present vs. absent) × 2 (implementation intention: present vs. absent) ANCOVA was conducted on the dependant variable of fitness center attendance at T2 controlling for fitness center attendance at T1. Estimated marginal means for gym attendance at T2 for the mental simulation and implementation intention (*M* = 10.15, *SE* = 0.44, 95% CI = 9.29, 11.02), mental simulation (*M* = 10.63, *SE* = 0.44, 95% CI = 9.77, 11.49), implementation intention (*M* = 10.49, *SE* = 0.43, 95% CI = 9.63, 11.34), and the control (*M* = 10.44, *SE* = 0.46, 95% CI = 9.54, 11.34) intervention groups suggested few differences (**Table 4**). This was corroborated by our analyses. We found no statistically significant main effects of mental simulation [*F*(3,180) = 0.02, *p* = 0.877, $\eta_{p}^{2}$ < 0.001] and implementation intention [*F*(3,180) = 0.24, *p* = 0.626, $\eta_{p}^{2}$ = 0.001], or a significant time × group interaction effect [*F*(3,180) = 0.36, *p* = 0.549, $\eta_{p}^{2}$ = 0.002], on fitness center attendance. We also conducted an analysis for the subsample that completed the 19-week follow-up measure at T3. A 2 × 2 ANCOVA with fitness center attendance at T1 as the covariate revealed no significant main effects of mental simulation [*F*(3,72) = 0.41, *p* = 0.524, $\eta_{p}^{2}$ = 0.006] and implementation intention [*F*(3,72) = 0.03, *p* = 0.868, $\eta_{p}^{2}$ = 0.000], or a significant time × group interaction effect, on fitness center attendance [*F*(3,72) = 0.06, *p* = 0.816, $\eta_{p}^{2}$ = 0.001].

**Table 4:** Results of the linear mixedmodel of the effects of the intervention on physical activity in Study 2.

Outcome measures	Control group (*n* = 43) Mean (*SD*)	Implementation intention group (*n* = 48) Mean (*SD*)	Mental simulation group (*n* = 47) Mean (*SD*)	Mental simulation plus implementation intention group (*n* = 46) Mean (*SD*)	*F*_mental simulation × implementation intention_	Effect size (main effect of implementation intention)^c^		Effect size (main effect of mental simulation)^d^		Effect size (interaction effect of mental simulation and implementation intention)^e^	
								
						*d*	95%CI^b^	*d*	95%CI^b^	*d*	95%CI^b^
Fitness center attendance (T1)	7.33 (4.93)	6.23 (4.37)	7.47 (5.01)	7.30 (5.62)							
Fitness center attendance (T2)	10.77 (7.04)	9.40 (5.91)	11.15 (7.48)	10.46 (7.96)	0.36	0.01	-0.36; 0.39	0.09	-0.29; 0.46	0.08	-0.30; 0.46
Fitness center attendance (T3)^a^	19.42 (12.18)	16.22 (11.16)	19.45 (11.89)	16.92 (10.70)	0.06	0.10	-0.35; 0.55	0.05	-0.40; 0.50	0.14	-0.38; 0.65


*T1, Time 1; T2, Time 2; T3, Time 3.*

*^a^Sample size for all groups, *n* = 20.*

*^b^95%CI = 95% confidence interval of the effect size.*

*^c^Effect sizes for the mental simulation and implementation intention group and implementation intention group vs. Mental simulation group and control group comparisons at T2 and T3 controlling for T1.*

*^d^Effect sizes for the mental simulation and implementation intention group and mental simulation group vs. Implementation intention group and control group comparisons at T2 and T3 controlling for T1.*

*^e^Effect sizes for the mental simulation and implementation intention group vs. Mental simulation group and implementation intention group and control group comparisons at T2 and T3 controlling for T1.*

### Discussion

The aim of this study was to test the unique and interactive effects of an intervention comprising motivational and volitional components on fitness center attendance. In contrast to Study 1, the intervention adopted mental simulation to target motivation and implementation intention to target volitional components in an appropriately powered, full-factorial, randomized-controlled design. Results revealed no main effects for either of the implementation intention or mental simulation intervention, or their interaction, on fitness center attendance participation at 6 weeks post-intervention. We observed identical results for the 19-week follow-up for the University of Essex fitness center participants. These results provide further evidence against the efficacy of mental simulations and implementation intentions, and a combination of the two, in changing physical activity behavior. Current data were based on a much larger sample size relative to Study 1 and used appropriate means to manipulate the intervention components consistent with the underlying theory and methods adopted in the original mental simulation (Pham and Taylor, 1999) and implementation intention (Orbell and Sheeran, 1998; Gollwitzer, 1999) studies. The study also adopted fitness center attendance an objective indicator of the physical activity behavior. In addition, we adopted a factorial design to compare the efficacy of each intervention component separately.

## General Discussion

The aim of the present research was to test the unique and combined effects of two theory-based motivational and volitional components to promote physical activity behavior: mental simulations and implementation intentions. The studies adopted experimental designs to examine the unique and interactive effects of these intervention components on self-reported physical activity (Study 1) and attendance at a fitness center (Study 2). Results of both studies indicated no statistically significant effects of the mental simulation and implementation intention interventions on physical activity behavior. As a consequence, we had to reject our primary hypothesis that the motivational (mental simulation) and volitional (implementation intention) components would lead to increased physical activity behavior relative to each of the components alone. We also rejected our hypothesis that each of the intervention components would have main effects on behavior with greater physical activity participation for participants receiving either of the intervention conditions relative to participants in the control group. We were not able to conduct mediation analyses with the self-efficacy and planning measures as mediating variables as there were no effects on the main outcomes in both studies, contrary to expectations. However, it is important to put these findings into perspective with respect to the limitations of the studies. Study 1, in particular, was conducted on a small sample and effect size statistics indicated medium-sized effects of the interventions, but the study was not sufficiently powered to detect effects of this size.

Current findings are inconsistent with trends in the literature if we compare our results with findings from studies that have investigated the unique and combined effects of mental simulation and implementation intention on health behavior. Meta-analyses of interventions examining effects of implementation intention on health behaviors have shown small-to-large effect sizes (Gollwitzer and Sheeran, 2006; Adriaanse et al., 2011; Bélanger-Gravel et al., 2013), but with substantive unresolved heterogeneity and a number of individual studies with similar designs have found null effects (Jackson et al., 2005, 2006; Skar et al., 2011; Huang, 2012; Scholz et al., 2013; Jessop et al., 2014). Similarly, research examining the effects of mental simulations has largely supported effects of the intervention, again, some studies report null findings. The combination of motivational and volitional components was supposed to facilitate the enactment of the behavior through maximizing motivation and facilitating implementation (Milne et al., 2002; Hagger and Chatzisarantis, 2014). In Study 1, it is possible that the intervention effects may have been effective and that the study had been inadequately powered. However, this possibility was not corroborated by the null findings and weak effects in Study 2. It is important to note that intervention studies combining both techniques have not always supported this pattern of effects. For example, Hagger et al. (2012b) found a significant effect of implementation intention on alcohol consumption but no effect for mental simulation or interaction effect of mental simulation and implementation intention, while Hagger et al. (2012a) demonstrated that mental simulation was significantly more effective for the same behavior with no effect for implementation intentions or the interaction.

It is important to note that the methods adopted in both of the current studies included variations to the conventional operationalization of the intervention manipulations. For example, we adopted slightly different versions of the mental simulation and implementation intention exercises across the studies. An ‘if-then’ format was used in Study 1 while a global format (a ‘what, when, and where’ plan) was adopted in Study 2. Some researchers (Chapman et al., 2009; Hagger and Luszczynska, 2014) have advocated the use of the ‘if-then’ format in implementation intention research to make the link between encountered situational cue and the behavior (Gollwitzer and Sheeran, 2006). However, there is, as yet, no definitive consensus on the appropriate format to adopt (Hagger et al., 2016) and few researchers have compared the moderating effect of plan format (e.g., ‘if-then’ format vs. the global format) on the effect of implementation intentions on health-related behaviors (Chapman et al., 2009; Pakpour et al., 2016). Despite these variations, there was no indication of a moderating effect of format in the current studies, as we found consistently null effects regardless of implementation intention format. As a consequence, we do not think that our findings can be dismissed on the basis of methodological variation.

Another factor that may have influenced current findings was the operationalization of the mental simulation manipulation. Two different mental simulation manipulations were used across the studies: a process mental simulation manipulation was used in Study 1 while an outcome mental simulation was used in Study 2. One possible explanation of the current findings is that neither of the simulation techniques was effective in promoting greater motivation. In previous studies, process simulations have been generally found to be more effective than the outcome simulations (Pham and Taylor, 1999). The greater efficacy of process simulations may have been due to the promoting greater self-efficacy through self-modeling and more positive attitudes toward the behavior through increasing knowledge of the regulation skills required. Outcome mental simulations, on the other hand may reflect ‘idealized’ outcome states which are ‘fantasy’-based and may be disruptive when presented alongside planning techniques like implementation intentions. Outcome simulations, for example, may distract from participants’ attention from the goal rather than to the behavior and the appropriate cues. However, while this may explain why the outcome simulations were not effective in the current study, it does not provide an explanation of the poor effectiveness of the process simulation alongside the implementation intentions in Study 1. One possibility is that because the mental simulation techniques, both process and outcome, were self-directed, the imagery did not focus on behaviourally relevant actions and cues that would lead to more effective behavioral engagement. There are other imagery-based techniques such as mental contrasting (Stadler et al., 2009; Adriaanse et al., 2010; Oettingen et al., 2013), which may help to implement the goal by bolstering motivation without distracting the attention to other goals (Knauper et al., 2009). Mental contrasting focuses on the reinforcement of the volitional phase of the Model of Action Phases and tap into personally relevant barriers rather than focusing on outcomes which may not be realistic or efficacious. Certainly further interventions may consider including ‘personally tailored visualization’ manipulations that focus the actor’s attention on the specific action (i.e., doing physical activity) and relevant cues to action.

### Limitations and Suggested Solutions

A key limitation of the current study was the relatively small sample sizes, particularly in Study 1. This would found to be particularly pertinent given that the effect size seemed to indicate possible medium-sized effects. Study 2 adopted a larger sample size and used similar, but not identical, methods but did not find any effects. It is important to note that in Study 2, we based our *a priori* sample size calculation on the implementation intention effect size only (Gollwitzer and Sheeran, 2006), rather than the interaction effect for the mental simulation component or the interaction of this component with implementation intentions. This means that the study was not powered to find the interaction effect. In addition, powering Study 2 to detect a small effect size, such as that found in Bélanger-Gravel et al.’s (2013) research, would have required a much larger sample. But, powering the study to find such a small effect size would mean to focus on an effect that would be of little practical significance. Interestingly, adhering to such an estimate would mean identification of a sample size that would exceed the largest sample size in the sample of studies included in Bélanger-Gravel et al.’s (2013) meta-analysis. Meaning that all studies testing for planning intervention effects in physical activity are underpowered. In reality, it seems that the true effect size derived from the current study was even smaller than this estimate. This means that the likelihood of finding a statistically significant effect size would require a very large sample size. This example, therefore, illustrates the imperative of an appropriate sample size, but also illustrates that, in some cases, a very small effect size would make it unfeasible to attain the required sample size. However, such studies would perhaps have limited value because they would be attempting to find an effect which has little or no practical significance.

The variability in effect sizes for these interventions is also an issue noted in other studies on implementation intentions. Focusing on implementation intentions, previous meta-analyses in physical activity, and across health behaviors more generally, have reported highly variable effect sizes ranging from small to large (Gollwitzer and Sheeran, 2006; Adriaanse et al., 2011; Bélanger-Gravel et al., 2013). Such variability points to the likely presence of moderator variables. This presents a problem when deciding which effect size should be used in statistical power analyses for new interventions and replications of implementation intention studies. There is, therefore, a real need to adopt standardized designs for the manipulation of intervention techniques like implementation intentions and mental simulations and conduct a systematic evaluation of the effect as a function of candidate moderators (Prestwich et al., 2015; Hagger et al., 2016). This is consistent with research in other areas of psychology advocating the adoption of standardized designs and large-scale, highly powered replications of intervention effects to allay problems associated with small samples and lack of statistical power (Earp and Trafimow, 2015; Hagger and Chatzisarantis, 2016).

A further limitation of the current research which may have influenced the findings is the definition of the physical activity provided for the participants and failure to prompt a goal intention. No formal definition of the regular physical activity was provided to participants. In Study 1, our self-report measure of physical activity, the IPAQ, provided a description of each level of intensity of physical activity but it was not defined prior to completing the manipulations. Further, participants in Study 1 were not provided with a specific goal intention to participate in more physical activity than they did before the trial. In Study 2, the WHO physical activity recommendations were introduced alongside mental simulation and implementation intention manipulations. Providing a specific definition may have served as a prompt for participants to form a goal to reduce physical activity when responding to the intervention manipulations. Nevertheless, a specific goal intention was not prompted, which may have stymied the effectiveness of the intervention.

Although we used a self-report measure of physical activity with good psychometric properties in Study 1 (Craig et al., 2003) and fitness center attendance as an objective proxy measure of physical activity in Study 2, both measures have limitations. Even though self-report measures have good concurrent validity with objective measures of physical activity, there is still the possibility of response bias. Fitness center attendance is also problematic as it only accounts for frequency of attendance and not the type, duration or intensity of the activities performed once there. These measures may, therefore, have led to bias in the reporting of behavior. The inclusion of a self-report measure of physical activity in Study 2 alongside the fitness center attendance measures would have provided additional indication of the types of physical activity behavior participants engaged in during their fitness center attendances. Replication of the current paradigm with ‘gold standard’ objective measures of physical activity like heart rate monitors and accelerometers would be required to unequivocally support the current null findings.

## Conclusion

Current findings do not support the effectiveness of the individual or combined use of mental simulations and implementation intention techniques to promote physical activity behavior. The studies had certain strengths, particularly Study 2 which included a factorial design and efforts to match of manipulations and stimuli with those adopted in previous studies. Even if we tried to closely mirror the suggested features of each intervention component in our manipulations, there are some salient deviations from other implementation intention and mental simulation studies in terms of the format adopted. The relatively small sample sizes, particularly that of Study 1, is a significant limitation. Notwithstanding these limitations, the current findings provide indication that these intervention techniques may not be as effective as expected or reported in previous meta-analyses. However, we cannot, of course, unequivocally rule out the possibility that changes in sample size and format may overturn the null findings, and we therefore look to further replications that refine our methods to provide further replication tests of these effects to confirm or reject our null findings.

## Author Contributions

CM, AG, BA, OF, and MSH were equally involved in the conception of the studies, the acquisition, analysis and interpretation of data, in drafting the manuscript and the final approval of the version to be published.

## Conflict of Interest Statement

The authors declare that the research was conducted in the absence of any commercial or financial relationships that could be construed as a potential conflict of interest.

## Footnotes

1 We adopted a 1-week follow-up in order to conduct a test of the concurrent validity of our self-report measure of physical activity using accelerometers worn by a subsample of the participants. However, we were not able to retrieve the data from the accelerometers due to equipment failure.

2 The majority of participants scored at least 3 (on a 6-point scale) on the manipulation check variables for mental simulation and implementation intention manipulation checks suggesting that the majority had sufficiently engaged in the intervention. However, a small minority reported the lowest score (1) on the mental simulation (*n* = 2) and implementation intention (*n* = 4) manipulation check variables suggested that they had not engaged. However, excluding these participants from the main analyses did not alter the effects with no main effect of groups on physical activity in MET-min per week at T2 and T3, taking into account physical activity at T1.

3 Similar to Study 1, we tested whether the mental simulation manipulation had an effect on the motivation by examining the effects of the intervention effects on intention. A univariate ANCOVA with mental simulation as fixed factor (present vs. absent) was conducted on the dependant variable of intention at T2 controlling for intention at T1. Yet, the analyses revealed no significant time × group interaction effect [*F*(1,182) = 0.02, *p* = 0.902, $\eta_{p}^{2}$ = 0.000] on intention.
